# Oroxylin-A Rescues LPS-Induced Acute Lung Injury via Regulation of NF-κB Signaling Pathway in Rodents

**DOI:** 10.1371/journal.pone.0047403

**Published:** 2012-10-10

**Authors:** Tzu-Ling Tseng, Mei-Fang Chen, Ming-Jen Tsai, Yung-Hsiang Hsu, Chin-Piao Chen, Tony J. F. Lee

**Affiliations:** 1 Institute of Medical Sciences, Tzu-Chi University, Hualien, Taiwan; 2 Institute of Pharmacology and Toxicology, Tzu-Chi University, Hualien, Taiwan; 3 Department of Life Science, Tzu-Chi University, Hualien, Taiwan; 4 Center of Vascular Medicine, College of Life Science, Tzu-Chi University, Hualien, Taiwan; 5 Department of Pathology, College of Medicine, Tzu-Chi University, Hualien, Taiwan; 6 Department of Research, Buddhist Tzu-Chi General Hospital, Hualien, Taiwan; 7 Department of Emergency Medicine, Buddhist Tzu-Chi General Hospital, Hualien, Taiwan; 8 Tzu-Chi College of Technology, Hualien, Taiwan; 9 Department of Chemistry, National Dong Hwa University, Hualien, Taiwan; 10 Department of Pharmacology, Southern Illinois University School of Medicine, Springfield, Illinois, United States of America; Johns Hopkins School of Medicine, United States of America

## Abstract

**Background and Purpose:**

Successful drug treatment for sepsis-related acute lung injury (ALI) remains a major clinical problem. This study was designed to assess the beneficial effects of post-treatment of oroxylin A (OroA), a flavonoid, in ameliorating lipopolysaccharides (LPS)-induced lung inflammation and fatality.

**Experimental Approach:**

Rats were injected with LPS (10 mg/kg, iv) to induce ALI, and OroA was given (15 mg/kg, iv) 1 hr or 6 hrs after LPS challenge. Twenty four hrs after LPS challenge, biochemical changes in the blood and lung tissues, and morphological/histological alterations in the lung associated with inflammation and injury were examined. Therapeutic effect of OroA was assessed by measuring the survival rate in endotoxemic mice.

**Key Results:**

LPS (10 mg/kg, iv) significantly altered WBC counts, elevated plasma tumor necrosis factor (TNF)-α and nitric oxide (NO), increased pulmonary edema, thickened alveolar septa, and decreased survival rate. These changes were ameliorated by OroA (15 mg/kg, iv) administered 1 hr or 6 hrs after LPS challenge. This post-treatment also significantly attenuated LPS-induced activation of nuclear factor-κB (NF-κB) and the release of high mobility group box 1 (HMGB1) in lung tissues. Furthermore, post-treatment with OroA (60 mg/kg, ip) administered 1 hr or 6 hrs after LPS challenge in mice significantly increased survival rate.

**Conclusion and Implication:**

OroA administered after induction of ALI by LPS significantly prevent and revere lung tissues injuries with increased survival rate. Positive post-treatment effects of OroA suggest that OroA is a potentially useful candidate for managing lung inflammation in LPS-induced endotoxemia and septic shock.

## Introduction

The acute respiratory distress syndrome (ARDS), an indication of acute lung injury (ALI), is highly associated with sepsis [1], [2] pneumonias [3], and severe acute respiratory syndrome (SARS) [3], [4]. Pathogenesis of ARDS involves release of proinflammatory mediators such as tumor necrosis factor (TNF)-α and nitric oxide (NO) [5]–[7] among others. High level of TNF-α production, an early warning signal, can aggravate lung injuries [8]. Activated alveolar macrophages and neutrophils also cause excessive productions of NO via the expression of inducible nitric oxide synthase (iNOS) [9]. The high mobility group box-1 (HMGB1), a late acting cytokine, also contributes to significant lung damages [10], [11], although HMGB1 may be expressed earlier in different disease models [12]. Patients with ARDS and the endotoxemic animals have an early increase of TNF-α and NO, and the late elevation of HMGB1 in the lung epithelium fluid and plasma [13]. Accordingly, these proinflammatory mediators and their upstream NF-κB signaling pathway may play critical roles in the pathogenesis of ALI. Blockade of TNF, NO, HMGB1 release may lessen the severity of LPS-induced ALI [11], [14].

ARDS is characterized by a diffused alveolar damage, formation of hyaline membranes, protein-rich edema fluid in the alveolar spaces, capillary injury, and disruption of the alveolar epithelium [1], [3], [4]. Most studies have demonstrated that pre-treatment with various candidate drugs such as non-steroidal anti-inflammatory drugs (NSAIDs), corticosteroids, and some natural products prevent and delay severe inflammatory responses [15]–[17]. Beneficial effects of pre-treatments with these drugs, however, do not assure their effectiveness for the acute management (i.e., post-treatment) of sepsis syndrome. In fact, post-treatment of these drugs do not significantly reduce lung injury [16], [17], and the mortality due to ARDS remains high [18].

Polyphenols which exhibit anti-inflammatory effects are major components in many traditional herbal remedies [19]. Oroxylin A (OroA), an active component of Scutellariae radix (Huang Qin), is a biphenolic compound [20]. It inhibits activated transcription factor NF-κB, leading to a reduction of lipopolysaccharides (LPS)-induced iNOS and cyclooxygenase-2 (COX-2) expression in RAW 264.7 cells [20]. These results suggest that OroA is a potentially useful therapeutic agent for treating acute lung injury induced by LPS.

We, therefore, investigated effects of the post-treatment of OroA on LPS-induced lung inflammation and fatality in rats and mice, respectively. Our findings indicated for the first time that post-treatment with OroA 6 hrs after LPS challenge significantly reduced the lung inflammation accompanied by a reduction of cytoplasmic NF-κB-targeting HMGB1, plasma TNF-α and NO, and pulmonary edema. This post-treatment also significantly increased the survival rate of LPS-challenged endotoxemic mice.

## Results

### Altered Circulating WBC and Plasma TNF-α in LPS-induced ALI in Rats

**Figure 1:**
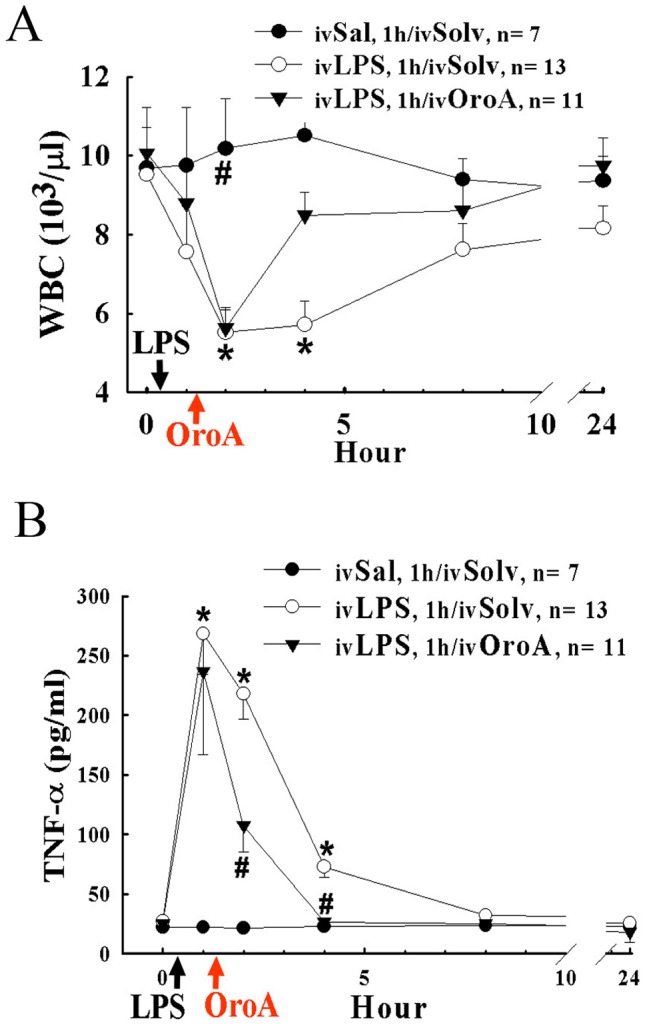
Effects of OroA on LPS-induced changes of the circulating WBC and plasma TNF-α level in the rats. OroA (15 mg/kg, iv) was administered 1 hr and examined 24 hrs after LPS treatment (10 mg/kg, iv) (Panels A and B). Normal saline (Sal) or Solvent (Solv, normal saline plus Tween 80 at 9∶1 ratio) was administered as controls. Black arrow indicates LPS treatment and red arrow OroA post-treatment. Data represent means±SEM. *P<0.05 indicates significant difference from the control, and #P<0.05 indicates significantly different from the LPS alone group. n indicates the number of experiments.

Changes of circulatory parameters, including WBC number and TNF-α, in rat models of LPS-induced acute lung injury (ALI) in the earlier (6 hr after LPS), middle (12 hr after LPS), and later (24 hr after LPS) stages were examined. An initial significant decrease in circulating WBC which peaked between the 2nd to 4th hr after LPS (10 mg/kg, iv) challenge was found (Fig. 1A). The WBC counts slowly returned to the basal level at 8th hr after LPS challenge. OroA (15 mg/kg, iv) administered 1 hr after LPS challenge prevented the decrease or facilitated the recovery of circulating WBC at the 4th hr after LPS administration. In contrast, plasma TNF-α was significantly elevated and peaked at the 1st hr, and was followed by rapid decline to the basal level at the 8th hr after LPS treatment (Fig. 1B). Administration of OroA (15 mg/kg, iv) 1 hr post LPS treatment significantly reduced the elevation of TNF-α in 1 hr (2 hrs after LPS challenge) with earlier decline to the basal level at the 4th hr after LPS treatment, and was maintained at this basal level 24 hrs after LPS challenge (Fig. 1B). OroA (15 mg/kg, iv) given at 6 hrs after LPS challenge, however, did not significantly altered LPS effects on circulating WBC or plasma TNF-α (data not shown). Hence, post-treatment of Oro-A was effective in reversing LPS-induced WBC reduction, and suppressing enhanced circulating TNF-α levels in LPS-induced ALI (Figs. 1A and B).

### OroA Post-treatment Ameliorated Thickened Intra-alveolar Septa in LPS-induced ALI

**Figure 2:**
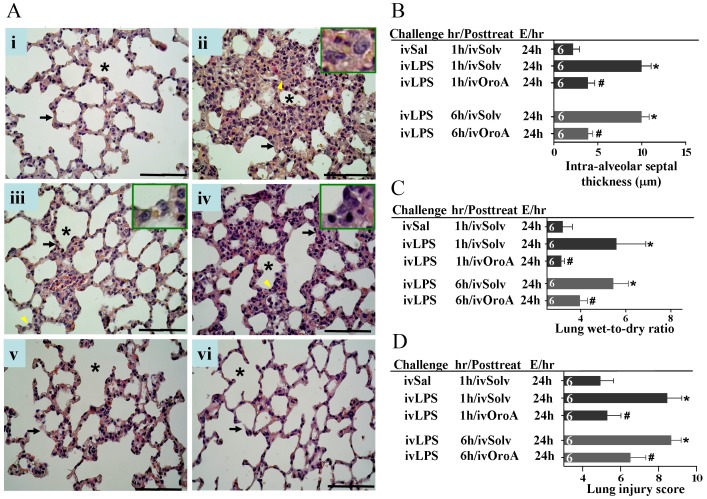
Inhibition of LPS-induced lung inflammation by OroA post-treatment. Representative sections of the rat lung tissues were stained with hematoxylin and eosin. In control section (panel Ai), normal alveoli (*asterisk*) and alveolar septa (*arrow*) with few neutrophils were shown. 24 hrs after LPS treatment (10 mg/kg, iv), thickened septa were observed in panel Aii, and inset is the enlarged area (indicated by arrowhead) of activated alveolar macrophages. Oro-A (15 mg/kg, iv) given 1 hr (panels Aiii and B) and 6 hrs (panels Aiv and B) after LPS treatment significantly reversed thickened septa when examined 24 hrs after LPS challenge. This concentration of Oro-A given 1 hr (panel Av) and 6 hrs (panel Avi) after saline (Sal) treatment did not show any effect when examined 24 hrs after LPS challenge. Panel C indicates that the enhanced lung wet-to-dry weight (W/D) ratio following LPS treatment (10 mg/kg, iv) was reversed by OroA treatment (15 mg/kg, iv) administered 1 hr or 6 hrs after LPS challenge. In panel D, LPS (10 mg/kg, iv) significantly increased lung injury score comparing to that of Sal (normal saline) control when examined 24 hrs (E/24h) after LPS challenge. The increase was reversed significantly by OroA (15 mg/kg, iv) administered 1 hr or 6 hrs after LPS challenge. Solv, normal saline plus Tween 80 at 9∶1 ratio; hr/Posttreat (post-treatment hour after LPS treatment); E/hr (examination hour after LPS challenge). Data are means±SEM. *P<0.05 indicates significant difference from the normal control (ivSal-ivSolv) group. #P<0.05 indicates significantly different from the respective (1 hr or 6 hrs) LPS alone (ivLPS-ivSolv) group. The number in each column represents the number of rats used. Scale bar, 50 µm.

In order to explore the therapeutic benefit of OroA, we examined if OroA post-treatment prevented LPS-induced pathological changes of lung tissues at the different stages of inflammation. In urethane-anesthetized rats, the intra-alveolar septa became significantly thicker (Figs. 2Aii and 2B) with accumulation of activated alveolar macrophages (inset in Fig. 2Aii) 24 hrs after LPS treatment (10 mg/kg, iv). Administration of OroA (15 mg/kg, iv) 1 hr after LPS challenge significantly reduced LPS-induced accumulation of activated alveolar macrophages (inset in 2Aiii) and attenuated thickened intra-alveolar septa (Figs. 2Aiii and 2B). Similar results were found when OroA (15 mg/kg, iv) was administered 6 hrs after LPS challenge (Figs. 2Aiv and 2B). OroA (15 mg/kg, iv) alone did not cause notable sign of pathological changes (Figs. 2Aiv and 2B).

### OroA Post-treatment Reduced the Increased Lung Wet-to-dry Weight Ratio in LPS-induced ALI

In order to examine whether Oro-A inhibited the enhanced vascular permeability and prevented lung inflammation induced by LPS, pulmonary edema formation assay was performed. LPS (10 mg/kg, iv) treatment significantly increased the lung wet-to-dry (W/D) weight ratio (Fig. 2C) when examined 24 hrs after LPS challenge. The increase was reduced significantly by OroA (15 mg/kg, iv) administered 1 hr and 6 hrs after LPS treatment (Fig. 2C).

### OroA Post-treatment Inhibited the Enhanced Lung Injury Scores in LPS-induced ALI

The severity of LPS-induced lung injury was further evaluated. The lung injury score was obtained based on the presence of cell infiltration and accumulation, pulmonary edema, and/or hemorrhage [21]. The lung injury scores of LPS-treated groups were significantly increased. The increase was significantly reduced following OroA treatment (15 mg/kg, iv) 1 hr and 6 hrs following LPS challenge (Fig. 2D).

### OroA Reduced the Elevated Plasma NO and iNOS Expression in the Lung Tissues of LPS-induced ALI

**Figure 3:**
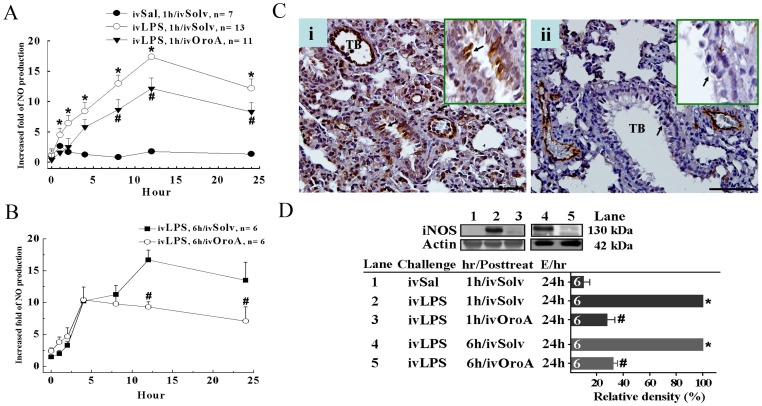
OroA inhibition of LPS-induced plasma NO and expression of iNOS in the lung tissues of the rats. Panel A indicates plasma NO concentrations in control (Sal followed by Solv), LPS (10 mg/kg, iv) followed by Solv-treated, and LPS followed by OroA-treated (15 mg/kg, iv) groups. OroA was administered 1 hr (panel A) or 6 hrs (panel B) and examined 24 hrs (E/24h) after LPS challenge. Data are means±SEM. *P<0.05 indicates significant difference from the normal control. #P<0.05 indicates significantly different from the LPS 1 hr or 6 hrs after LPS challenge. n indicates the number of experiments. In panel Ci, iNOS immunoreactivities (dark brown color) in rat terminal bronchioles (TB) and clara cells (inset of enlarged area indicated by arrows in Fig Ci and ii) were significantly expressed 24 hrs after LPS (10 mg/kg, iv) treatment. The expression was inhibited by OroA (15 mg/kg, iv) administered 6 hrs and examined 24 hrs (E/24h) after LPS challenge (panel Cii). The enhanced iNOS protein production induced by LPS (Lane 2 in panel D) and its inhibition by OroA (15 mg/kg, iv) administered 1 hr (Lane 3 in panel D) or 6 hrs after LPS (lane 5 in panel D). Actin levels were served as internal control. The results are summarized as the relative density (%) of iNOS production. hr/Posttreat (post-treatment hour after LPS treatment); E/hr (examination hour after LPS challenge). Data are means±SEM. *P<0.05 indicates significant difference from the control (Sal followed by Solv, Lane 1 in panel D). #P<0.05 indicates significant difference from the respective LPS alone (ivLPS-ivSolv) group (Lanes 2 and 4 in panel D). Scale bar = 20 µm. The number in each column represents the number of rats examined.

Plasma NO concentration was significantly increased after LPS administration (10 mg/kg, iv) in anesthetized rats (Figs. 3A and 3B). The increase was significantly reduced by OroA (15 mg/kg, iv) administered 1 hr (Fig. 3A) or 6 hrs (Fig. 3B) after LPS challenge. Furthermore, 24 hrs after LPS treatment (10 mg/kg, iv), iNOS was significantly expressed in small conducting bronchioles and clara cells (inset in Fig. 3Ci). Also, numerous iNOS-positive macrophages were found in intra-alveolar septa (Fig. 3Ci). The expression was significantly inhibited by OroA (15 mg/kg, iv) administered 6 hrs after LPS challenge (Fig. 3Cii). Similarly, immunoblotting showed that iNOS production examined 24 hrs after LPS challenge was significantly suppressed by OroA (15 mg/kg, iv) administered 1 hr or 6 hrs after LPS challenge (Fig. 3D).

### OroA Inhibited the HMGB1 Release in the Lung Tissues of LPS-induced ALI

**Figure 4:**
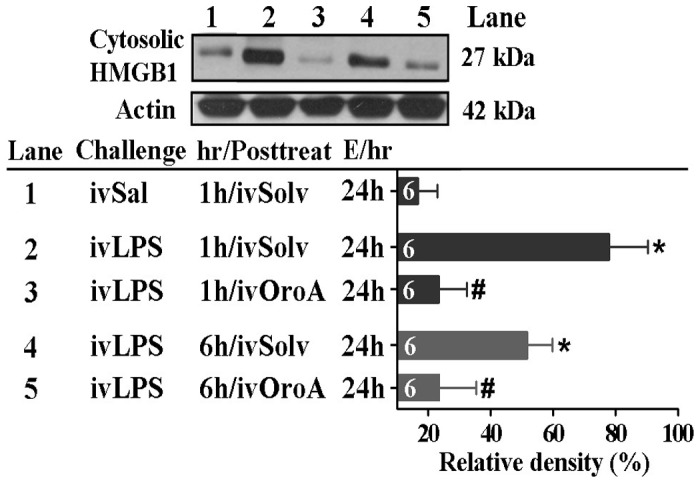
OroA attenuation of LPS-induced HMGB1 release in lung tissue. Comparing to Sal (saline) control (Lane 1), LPS (10 mg/kg, iv) significantly enhanced nuclear HMGB1 release into cytosolic fraction in lung tissue. The increased release was inhibited by OroA (15 mg/kg, iv) administered 1 hr (Lane 3) or 6 hrs (Lane 5) after LPS (Lane 4) challenge. The relative percent of cytosolic HMGB1 protein release was normalized to actin which served as an internal control, and summarized as the relative density (%) of cytosolic HMGB1 expression. Solvent (Solv, normal saline plus Tween 80 at 9∶1 ratio); hr/Posttreat (post-treatment hour after LPS treatment); E/hr (examination hour after LPS challenge). Data are means ± SEM. *P<0.05 indicates significant difference from the normal control (Lane 1). #P<0.05 indicates significant difference from LPS alone (ivLPS-ivSolv in Lanes 2 and 4). The number in each column represents the number of rats examined.

HMGB1, a later phase cytokine, is released from nuclei into the cytoplasmic region following LPS challenge [11], [12]. We therefore examined if OroA inhibited LPS-induced HMGB1 release. The concentration of cytoplasmic HMGB1 was relatively low in control and non-LPS-treated lung tissues but was significantly elevated 6 and 24 hrs after LPS (10 mg/kg, iv) challenge (Fig. 4). The elevated level at 24th hr was significantly reduced by OroA (15 mg/kg, iv) administered 1 hr or 6 hrs after LPS challenge (Fig. 4).

**Figure 5:**
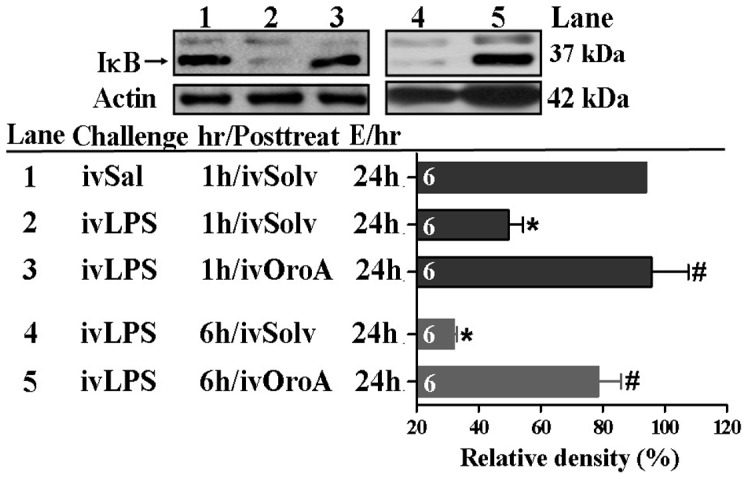
OroA inhibition of LPS-induced IκB degradation in lung tissue. Comparing to control group (Sal followed by Solv-treatment in Lane 1), LPS treatment (10 mg/kg, iv) significantly caused IκB degradation (Lanes 2 and 4). This effect, examined 24 hrs (E/24h) after LPS challenge, was prevented by OroA (15 mg/kg, iv) administered 1 hr (Lanes 3) or 6 hrs (Lane 5) after LPS challenge. The relative percent of total IκB was normalized to actin which served as an internal control, and was summarized as relative density (%). Sal (Saline); Solvent (Solv, normal saline plus Tween 80 at 9∶1 ratio); hr/Posttreat (post-treatment hour after LPS treatment); E/hr (examination hour after LPS challenge). Data are means±SEM. *P<0.05 indicates significant difference from the control (Lane 1). #P<0.05 indicates significant difference from the LPS alone group (Lanes 2 and 4). The number in each column represents the number of rats examined.

### OroA Inhibited LPS-induced NF-κB Activation and IκB Degradation in the Lung Tissues

**Figure 6:**
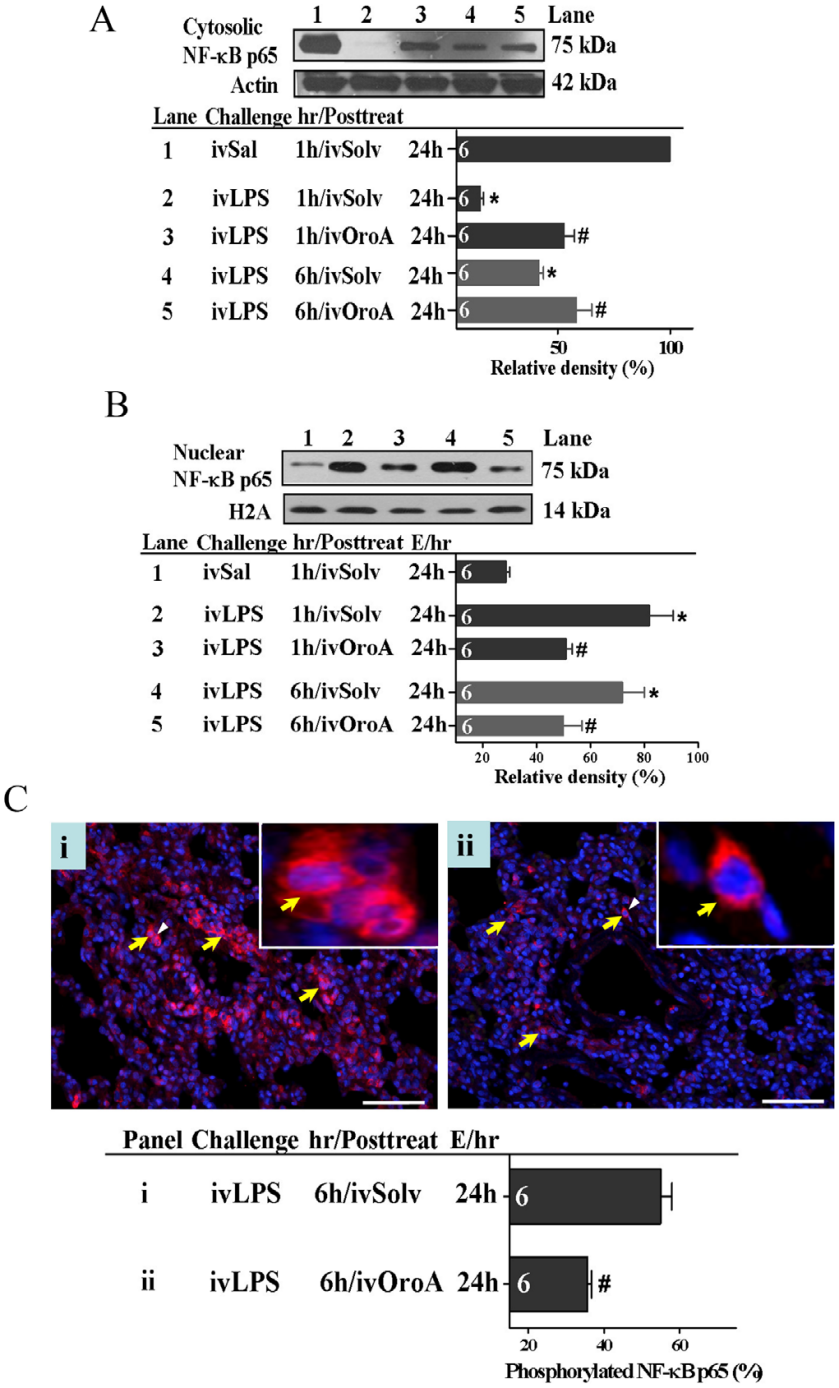
OroA inhibition of LPS-induced NF-κB activation in lung tissue of the rats. LPS treatment (10 mg/kg, iv) significantly decreased cytosolic NF-κBp65 expression in lung tissue (Lane 2 in Fig. 6A) comparing to that by Sal (Saline) followed by Solv (Solvent) treatment (Lane 1 in Fig. 6A). The reduction examined (E) 24 hrs after LPS challenge (Fig. 6A) was significantly reversed to similar extent by treatment with OroA (15 mg/kg, iv) administered 1 hr (Lane 3 in Fig. 6A) or 6 hrs (Lane 5 in Fig. 6A) after LPS. Bar graphs summarized the relative density (%) of cytosolic NF-κB protein that was normalized to actin (Fig. 6A). In parallel, LPS treatment (10 mg/kg, iv) significantly increased nuclear p65 expression (Lanes 2 and 4 in Fig. 6B) comparing to that by Sal followed by Solv treatment (Lane 1 in Fig. 6B). The increase examined 24 hrs after LPS challenge was significantly reversed to similar extent by treatment with OroA (15 mg/kg, iv) administered 1 hr (Lane 3 in Fig. 6B) or 6 hrs (Lane 5 in Fig. 6B) after LPS challenge. Bar graphs summarized the relative density (%) of nuclear NF-κB protein that was normalized to H2A. Solvent (Solv, normal saline plus Tween 80 at 9∶1 ratio); hr/Posttreat (post-treatment hour after LPS treatment); E/hr (examination hour after LPS challenge). Data are means ± SEM. *P<0.05 indicates significant difference from the normal control (Sal followed by Solv, Lane 1 in panels A and B). #P<0.05 indicates significant difference from the respective LPS treated group (LPS followed by Solv) (Lanes 2 and 4 in Figs. 6A and 6B) when examined 24 hrs after LPS challenge. The number in each column represents the number of rats examined. Fig. 6Ci indicates effects of LPS (10 mg/kg, iv) administered 6 hrs after LPS challenge on phosphorylated p65 cells in red (indicated by yellow arrows in panels Ci and Cii) which were examined 24 hrs (E/24h) after LPS challenge. Phosphorylated NF-κB p65 cells were significantly reduced by OroA (15 mg/kg, iv) administered 6 hrs after LPS challenge (panel Cii). Phosphorylated NF-κBp65 cells were calculated from randomly selected 4 fields with a total of 200 cells. Insets are enlarged areas indicated by white arrowheads denoting phosphorylated NF-κBp65 translocated into the nuclei. The nucleus in blue color was stained with DAPI. Data are means±SEM. #P<0.05 indicates significantly different from the LPS plus Solv-treated group. Scale bar, 20 µm. The number in each column represents the number of rats examined.

We further examined whether OroA inhibition of LPS-induced HMGB1 release was mediated by inhibiting transcriptional factor NF-κB activation and translocation. When examined at 24 hrs after LPS (10 mg/kg, iv) challenge, cytoplasmic IκB decreased significantly (ie., enhanced degradation) in lung tissues comparing to that in Sal-Solv treated control group (Fig. 5). This decrease was significantly reversed by OroA (15 mg/kg, iv) administered 1 hr or 6 hrs after LPS treatment (Fig. 5). In parallel, activated NF-κB was translocated from the cytoplasm into the nucleus in LPS-treated group, leading to a decreased cytosolic NF-κB (Fig. 6A) and an increased NF-κB in the nucleus (Fig. 6B). This translocation examined at 24 hrs after LPS treatment was inhibited equally by OroA (15 mg/kg, iv) given 1 hr and 6 hrs after LPS challenge (Figs. 6A and 6B). Furthermore, LPS treatment caused significant phosphorylation of NF-κBp65 (Fig. 6C-i). The phosphorylated NF-κBp65 was significantly inhibited by Oro-A (15 mg/kg, iv) given 6 hrs after LPS treatment (Fig. 6C-ii).

### OroA Improved the Survival Rate of LPS-induced ALI in Mice

**Figure 7:**
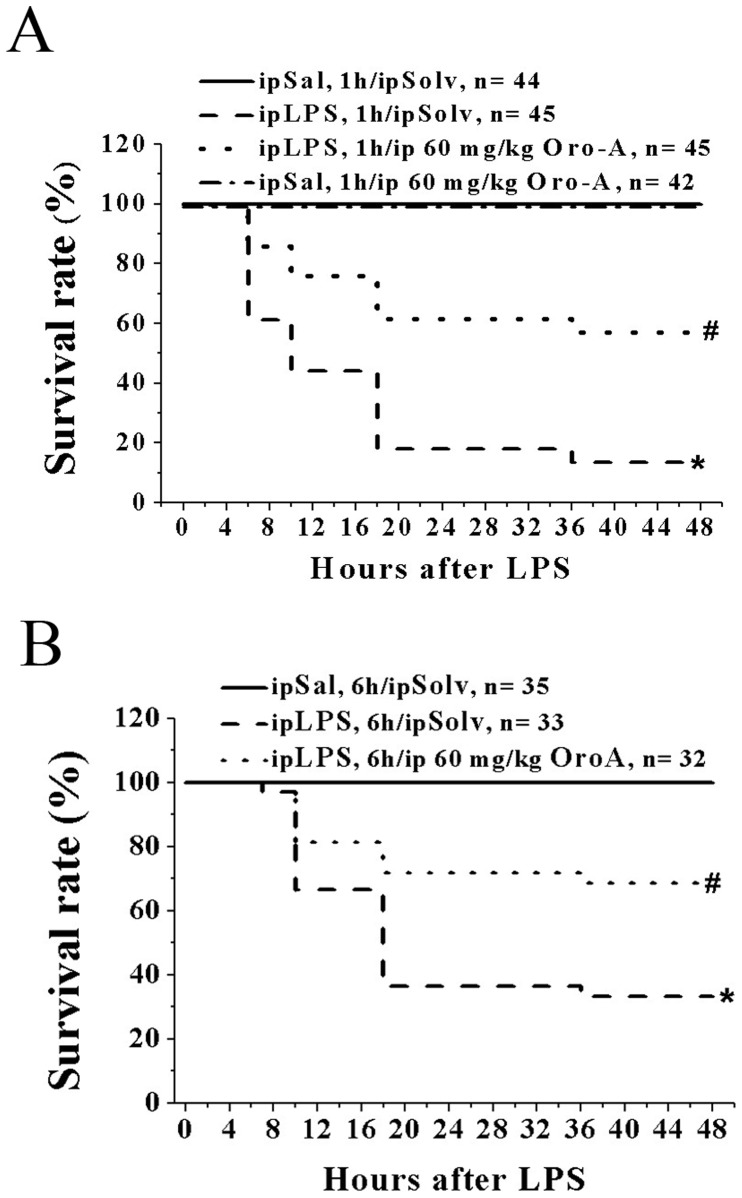
OroA improved the survival rate of endotoxemic mice. LPS treatment (100 mg/kg, ip) significantly decreased the survival rate in B6 mice (Panel A and B). The decrease was prevented by Oro-A (60 mg/kg, ip) administered 1 hr (Panel A) or 6 hrs (Panel B) after LPS challenge. Mice received Sal followed by Solv (ip) and Sal followed by OroA (60 mg/kg, ip) served as controls. *P<0.05 and #P<0.05 indicate significant difference from the control (Sal followed by Solv) group and LPS followed by Solv group, respectively. n indicates the number of experiments.

Survival rate, which is a key indication of therapeutic benefit of OroA, was examined in mice according to reports by others [17]. Mice were given lethal dose of LPS (100 mg/kg, ip) to induce endotoxemia. Less than 20% of the LPS-treated mice survived for 48 hrs (Figs. 7A and 7B). The survival rate of different mouse strains was not affected by 30 mg/kg OroA (ip) administered 1 hr after LPS challenge (results not shown) but was significantly improved by 60 mg/kg OroA (ip) administered 1 hr (Fig. 7A) or 6 hrs (Fig. 7B) after LPS challenge.

## Discussion

In the present study, we demonstrated that post-treatment with OroA significantly attenuated the lung inflammation and improved the survival rate of LPS-induced ALI in rodents. The beneficial effects of OroA involved the suppression of NF-κB signaling pathway, and the blockade of elevated circulating TNF-α and NO, release of the nuclear HMGB1 into the cytoplasm, accumulation of the macrophages in the interstitial space, and the thickened intra-alveolar septa of the lungs.

LPS-induced lung injury in the rat is frequently used as a model for studying the ALI [22]. The present findings of a large number of macrophages and PMNs in the lung tissues, increased endothelial permeability and tissue damage, and thickened intra-alveolar septa with excessive production of inflammatory cytokines and edema in the lungs are consistent with those reported by others [23]. Furthermore, the urethane-anesthetized rat model of ALI used in the present study has its advantage that hemodynamic changes can be monitored continuously for 24 hrs. The possibility, however, remains that beneficial effects of OroA may be attributed to urethane [24], [25] like other anesthetics such as ketamine and propofol which also have been reported to exhibit protection against LPS-induced endotoxemia [26], [27]. Although urethane at higher concentration (>1.1 g/kg, ip) exhibited anti-inflammatory effects and protected rats against lethal endotoxemia with reduced TNF-α release, urethane at lower concentrations (0.1 and 0.5 g/kg, ip) [25] did not show significant protection or prevent death from LPS challenge. The present results also indicated that urethane (0.5 g/kg, ip) alone did not suppress LPS-induced productions of iNOS, NO and the late acting cytokine HMGB1, or severe lung inflammation. Similar results were found in a conscious rat model (un-anesthetized rats) (our unpublished results). These results suggest that LPS-induced pathological changes are not significantly affected by urethane in the concentration used in the present study. For these reasons, the protective effect of OroA in urethane-anesthetized rats is not likely due to urethane at 0.5 g/kg (ip). For the same reasons, urethane at 0.5 g/kg (ip) does not seem to work in an additive or synergistic fashion with OroA, thereby exaggerating the natural ability of the compound. These results strongly support that the protective effect of OroA in urethane (0.5 g/kg, ip)-anesthetized rats is due primarily to OroA.

LPS stimulates the migration of circulating WBC and neutrophils into the sites of infection, promoting a rapid regulation of the immune response [28]. Our present results indicated that after LPS challenge, the circulating WBCs were significantly decreased initially followed by a return toward normal range at the later stage of inflammation. These results are consistent with those reported by others [29], and were significantly reversed by OroA administration (iv). Similar results were obtained from intraperitoneal administration of OroA (our unpublished data). Although these effects of OroA on LPS-induced changes of WBC per se remain to be determined, these data indicate that OroA preparation is bioavailable after iv and ip administrations.

LPS also activates the resident alveolar macrophages and neutrophils, causing inflammation and ALI [1]. In the present study, we found that the interstitial space was filled with activated alveolar macrophages and neutophils following LPS challenge. These pathological changes were reversed by OroA post-treatments, suggesting that OroA may inhibit LPS-induced leukocyte rolling and transmigration into the lung tissues. In addition, significant lung injuries including edema formation, thickened intra-alveolar septa, and enhanced lung injury scores were reduced significantly by OroA (15 mg/kg, iv) administered 1 hr or 6 hrs after LPS challenge. These results provide evidence supporting that OroA post-treatment is effective in reversing LPS-induced lung injuries.

We further examined whether Oro-A is effective in the later phase of lung inflammation, during which more inflammatory cascades are activated. Oro-A (iv, 15 mg/kg) administered 6 hrs after LPS treatment also significantly lowered activated macrophages accumulation and sequestration, suppressed pulmonary edema formation, and prevented the increase in septa thickness. These findings suggest that OroA is effective in ameliorating the late stage lung injury induced by LPS. This is supported further by the findings that LPS-induced expression of HMGB1, a semi-late (expressed 4 hrs after LPS challenge), and late acting proinflammatory cytokine [13], [30] which contributes to endotoxin-induced ALI and sepsis-associated lethality [10], [11], [13], was significantly inhibited by OroA (iv) administered 1 hr or 6 hrs after LPS challenge.

Vascular endothelial injury plays a critical role in the onset of ALI, which is characterized by disruption of the alveolar-capillary interface and promoting PMNs and inflammatory exudates to enter the alveoli [31]. In the lung inflammation, proinflammatory factors may impair the homeostasis of lung microvascular permeability, which is a prerequisite for PMNs sequestration in the lung [32]. In the present study, we demonstrated that OroA post-treatment (iv) not only suppressed sequestration of PMNs, but also attenuated formation of pulmonary edema. OroA appeared to relieve edema by protecting endothelial function to alleviate neutrophil sequestration. In addition, LPS-induced overproduction of TNF-α and NO can cause vascular endothelial cell injury and increased vascular permeability in the lungs [33]. These effects of LPS were prevented by intravenous OroA. These results provide additional evidence supporting that OroA exhibits potent anti-cytokine effects and is beneficial in preventing vascular endothelial injury during ALI.

NO, a potent vasodilator generated from L-arginine catalyzed by iNOS, is produced in activated alveolar macrophages by LPS stimulation [6], [7]. Overproduction of NO induced by LPS facilitates the progression of lung inflammation [2], [34]. In anesthetized models of ALI used in the present study, the plasma NO was increased after LPS challenge, and the increase was significantly reversed by OroA (15 mg/kg, iv) administered 1 hr or 6 hrs after LPS challenge. This is consistent to our previous report that OroA blocks iNOS gene expression and suppresses iNOS-catalyzed NO production in macrophages [20]. This is supported further by results of the present iNOS-immunohistochemistry and iNOS protein assay that LPS induction of iNOS in neutrophils, clara cells, and lung resident alveolar macrophages was prevented by OroA (15 mg/kg, iv) post-treatment. These results further support the anti-inflammatory effect of OroA.

Our present results that the WBC number in OroA-treated rats returned almost to the control level 5 hours after LPS, suggesting that OroA regulation of the circulating WBC is early and transient. This OroA effect, however, is similar to the transient effect of LPS on the circulating WBC. Furthermore, 6 to 24 hrs after LPS (Fig. 3), the increased NO and iNOS production was still markedly suppressed by OroA, while the circulating WBC returned to normal level. OroA appears to possess multi-sites of action in the early and the later phases of endotoxemia. Thus, OroA exhibited beneficial effect in managing endotoxemia early after application, but the effect was not transient. Strategically, it is logical to administer OroA as early as possible to block the early events of inflammation after LPS challenge. This may decrease the subsequent release of cytokines and development of irreversible damages such as due to release of HMGB1.

It is well established that the LPS-induced immunological cascade responses are complicated [1]–[4]. Although TNF responses following LPS-treatment were early and transient, it was possible that TNF initiated the cascade events [5], [35] before its return to the basal level. This was evidenced by the increased productions of NO, iNOS, and the late acting cytokine HMGB1. It is, however, difficult to claim that TNF is or is not responsible for mediating the pathogenesis of lung injuries in this animal model, since we did not use recombinant TNF fusion protein [35]or other approaches to block TNF activities. Regardless, the present studies demonstrated that OroA possessed multi-sites of action in the early and the later phases of endotoxemia.

NF-κB is a key transcription factor in response to host defense against infection [36]. Under unstimulated condition, NF-κB is present in the cytosol and is bound to inhibitory protein IκB [37], [38]. Following LPS challenge, NF-κB is translocated into the nucleus to drive expression of a variety of inflammatory genes, which are involved in the pathogenesis of ALI [39]. Therefore, blockade of NF-κB activation is expected to attenuate the ALI [10], [36]. This is supported by results of the present study demonstrating that OroA post-treatment inhibited the NF-κB activation and the release of NO and HMGB1. This is consistent with the notion that OroA prevention of LPS-induced NO and HMGB1 release is via its anti-NF-κB activity [20], [36].

The survival rate of experimental animals is an important indication for evaluating possible therapeutic benefit of potential drugs. We found that about 80% of mice died between 24 and 48 hrs after receiving the lethal dose of LPS; a result similar to that found by others [35]. The fatality of mice, however, was significantly reduced after animals were given OroA (60 mg/kg, ip) 1 hr or 6 hrs after LPS treatment, suggesting that OroA is a useful candidate for managing endotoxemia and septic shock.

Most studies have demonstrated in animal model of sepsis and lung injury that corticosteroids, baicalein (a flavonoid), and ethyl pyruvate when given simultaneously with or before LPS-induced experimental insult protect animals from multiple organ dysfunction and decrease mortality rate [15]–[17]. The beneficial effects of post-treatments of these drugs for ARDS in experimental animals and patients at high risk, however, are not reported. In addition, all these drugs do not significantly prevent the thickening of intra-alveolar septa or inhibition of monocytes migration at the later stages of lung injury. In clinical practice, high dose of corticosteroid (10 mg/kg) does not suppress the infiltration of inflammatory leukocytes, the production of cytokines, or the development of fibrotic changes in the lung [16]. In fact, patients who receive corticosteroids have increase collagen deposition with increased lung injury [16], suggesting that corticosteroids are not clinically effective when administered at the later phase of lung injury [16].

In summary, OroA post-treatment significantly attenuated the LPS-induced lung injuries with significantly increased survival rate of the endotoxemic animals. OroA amelioration of LPS-induced ALI involved inhibition of activated NF-κB and blockade of release of early and late-acting cytokines. OroA also inhibited the expression of cyclooxygenase 2 (COX-2) [20] which is known to play a role in lung inflammation [13]. Furthermore, our preliminary studies demonstrated that post treatment of OroA significantly reversed systemic hypotension with improved cardiac inotropy in LPS-treated rats. OroA, therefore, is a potentially useful compound that provides new strategy in clinical prevention and treatment of ARDS in sepsis.

## Materials and Methods

### Animal Source

Male Sprague-Dawley rats weighing 300–350 g and C57BL/6J (B6) mice weighing 25–35 g maintained under standard conditions at Tzu Chi University’s Animal Center were used. All experimental procedures were approved by the Animal Care and Use Committee of Tzu Chi University.

### Experimental Procedures

Rats were anesthetized with intraperitoneal (ip) urethane (0.5 g/kg, purchased from *Sigma-Aldrich Chemical, St Louis, MO*) [17]. Left and right femoral arteries were cannulated for recording blood pressure/heart rate and administering experimental drugs/collecting blood samples, respectively. Animals were allowed to stabilize for 60 min. Lipopolysaccharides *serotype 0127:B8* (*Escherichia coli* LPS, *Sigma-Aldrich Chemical*) was dissolved in sterile physiological saline immediately before use. All invasive procedures were operated under aseptic conditions. After LPS administration, animals were monitored for changes in mean arterial pressure (MAP) and heart rate (HR) for 24 hrs.

Animals were divided randomly into 5 groups: (1) *ivSal, 1h or 6 h/iv/Solv (Control) group*: 1 or 6 hrs after iv injection of normal saline (Sal), animals were administered solvent (Solv) (Sal plus Tween 80 at 9∶1 ratio) (iv), (2) *ivLPS, 1h/iv/Solv group*: 1 hr after receiving LPS (10 mg/kg, iv), animals were administered Solv (iv), (3) *ivLPS, 1h/ivOroA group*: 1 hr after LPS (iv) challenge, animals were administered OroA (15 mg/kg, iv), (4) *ivLPS, 6h/ivSolv group*: 6 hr after LPS (iv) challenge, animals were administered Solv (iv), and (5) *ivLPS, 6h/ivOroA group*: 6 hrs after LPS (iv) challenge, animals were administered OroA (15 mg/kg, iv). Twenty four hours after LPS challenge, animals in each group were sacrificed under anesthesia, and various tissues were removed and examined. Supernatants of blood samples also were collected and frozen at −80°C for subsequent assay of TNF-α and NO.

### WBC Measurement

Immediately after drawing, WBC in the whole blood samples (100 µL, containing 50 U/ml heparin) were counted with an automatic multi-parameter blood cell counter (*model Sysmex KX-21, Hyogo, Japan*) [40].

### Plasma TNF-α Measurement

Plasma TNF-α concentrations were measured by enzyme-linked immunosorbent assay (*rat TNF-α BMS622, Bender MedSystems, Vienna, Austria*) [41] according to manufacturer’s instruction.

### Plasma NO Measurement

Plasma NO was measured by Griess reaction [20]. Frozen plasma samples were thawed and deproteinized by incubation with 95% ethanol (*Sigma Chemicals*) at 4°C for 60 min, and centrifuged for 10 min at 12,000 rpm. One hundred µl of supernatant sample was incubated for 5 min with 100 µl Griess reagent (containing 0.1% N-1-napthylethylenediamine dihydrochloride, 10% sulfanilamide in 5% phosphoric acid, from *Sigma Chemicals*) in 96-well plates. Standard nitrite salt (*Sigma Chemicals*) solutions (0–100 µM) were used. Absorbance of reaction product was measured at 550 nm. Plasma nitrite concentrations (µM) were calculated from the standard curve.

### iNOS Immunohistochemistry (IHC)

The lung tissues were removed, dehydrated and embedded in paraffin, cut into 2-µm-thick sections and processed for IHC [33]. Sections were incubated in antigen retrieval buffer containing 10 mM Tri-sodium citrate, 0.05% Tween 20, pH 6.0 for 20 min, washed with phosphate-buffer saline (PBS), and permeablized with 0.1% Triton X-100 in PBS for 30 min. Nonspecific staining was blocked with normal serum blocking reagent (*Biogenx, San Ramon, CA*) for 30 min. This was followed by incubation with anti-iNOS mouse antibody (*BD Bioscience*, *NJ, USA,* 1∶200) overnight at 4°C. Super enhancer solution (*Biogenx, San Ramon, CA*) was added for 40 min, and a poly horseradish peroxidase conjugated anti-mouse IgG reagent was applied for 60 min followed by avidin-biotin-peroxidase complex and the substrate 3,3′-diaminobenzidine (DAB). Cell nuclei were counter-stained with hematoxylin for 30 sec. 6 to 9 sections were obtained from each tissue block per animal from 6 animals, and 1 section per animal was randomly selected for statistical analysis. All sections were mounted with a water-soluble mounting medium and examined under a light microscope (*Leica, Leica Microsystems, Wetzlar, Germany*) in a single blinded fashion.

### NF-κBp65 Immunofluorescence (IF)

Standard IF techniques were used [33]. Briefly, 2-µm paraffin-embedded tissue sections from the lungs were processed through antigen retrieval buffer, permabilized with Triton X-100 for 30 min, blocking of non-specific interaction with normal serum (*Biogenx*) for 1 hr, followed by washing and reaction with mouse phospho-NF-κB p65 (Ser536) monoclonal antibody (*Cell Signaling,* 1∶50) and anti-mouse IgG, Hilyte Fluor 555-labeled antibody (1∶200, *AnaSpec*). Cell nuclei were counter-stained with DAPI (1∶200, *KPL, Washington D.C, USA*) for 20 min. 6 to 9 sections were obtained from each tissue block per animal from 6 animals, and 1 section per animal was randomly selected for statistical analysis. All sections were mounted with a water-soluble mounting media and examined under a fluorescence microscope (*Leica, Leica Microsystems, Wetzlar, Germany*).

### Isolation of Nuclear and Cytosolic HMGB1 and NF-κB Proteins

Frozen lung tissues were homogenized in a buffer containing PBS and phosphatase inhibitors (*Nuclear extract kit, Active motif, Carlsbad, CA*) [4], and centrifuged at 500 rpm for 5 min. The resulting pellets were resuspended in 500 µl hypotonic buffer and incubated for 15 min. Nuclei were isolated by centrifugation at 10,000 rpm for 1 min. The supernatants that contained cytoplasmic and membrane proteins were collected and stored at −80°C. The pellets were resuspended in 50 µl complete lyses buffer, and nuclear extracts were recovered by centrifugation at 10,000 rpm for 10 min. The supernatants were collected and stored at −80°C for immunoblotting.

### Immunoassay

Protein concentrations were quantified with bicinchoninic acid (BCA) protein assay reagent (*Pierce chemicals, Rockford, IL*). Aliquots of the extracts were diluted in a 1∶4 ratio with sample buffer (0.25 M Tris-Cl pH 6.8, 2% 2-mecaptoethanol, 8% sodium dodecyl sulfate [SDS], 0.02% bromophenol blue, and 40% glycerol) and boiled for 5 min at 100°C. All protein extracts were quantitated simultaneously to ensure subsequent equal protein loading. Protein extracts were separated on 10% SDS polyacrylamide gels (20 µg of protein per lane) and transferred onto a polyvinylidene difluoride membrane (*Bio-Rad, Hercules, CA*) by semidry electroblotting (*Amersham Biosciences, Buckinghamshire, UK*) for 45 min. Blots were blocked for 2 hrs at room temperature with 5% non-fat milk in Tris-buffered saline with 0.25% Tween 20, and then washed in TBS-T buffer solution. Membranes were hybridized with the mouse iNOS monoclonal IgG (1∶500, *BD Transduction, California, USA*), mouse HMGB1 monoclonal IgG (1∶1000, *Abcam, Cambridge, UK*), mouse IκB monoclonal IgG (1∶1000, *Santa Cruz Biotechnology, California, USA*), and rabbit NF-κB monoclonal antibodies (1∶500, *Santa Cruz Biotechnology*) in immunoreaction enhancer solution (*Toyobo, Osaka, Japan*) at 4°C for overnight. After wash, blots were exposed to horseradish peroxidase-conjugated anti-mouse IgG and anti-rabbit secondary antibodies (1∶2000, *KPL, Washington D.C, USA*) for 1 hr at room temperature and immunoreactivities were visualized using an enhanced chemiluminescence (ELC) detection system (*PerkinElmer life science, Massachusetts, USA*). After stripping, blots were reporbed with a mouse anti-actin antibody (1∶4000, *Chemicon, Illinois, USA*) and rabbit histone H2A polyclonal (1∶2000, *Cell Signaling technology, Beverly, CA*) for normalization to equal protein loading. After scanning blots into computer, individual bands were analyzed by Image J software *(*National Institute of Mental Health*).*

### Measurement of Pulmonary Edema

The right lungs were removed and the wet weights were obtained. Lung tissues were weighed again 3 days after drying at 55°C. The wet-to-dry (W/D) ratio was calculated as follows: W/D ratio =  (wet weight-dry weight)/dry weight [14].

### Histopathology

Lung specimens were fixed in 4% paraformaldehyde (pH 7.6, *Sigma Chemicals*) overnight at room temperature [42]. After washing with tap water for 10 min, specimens were dehydrated through a graded series of ethanol (75% for 30 min, 80% for 60 min, 95% for 3 hr, and 100% for 30 min, from *Sigma Chemicals*) and embedded in paraffin. Two µm sections were placed on glass slides and dried at 37°C overnight. The slides were deparaffinized in non-xylene solution (3 times × 5 min per each time). After rehydration (ethanol at 100% twice for a total of 6 min, at 95% for 1 min, and at 75% for 1 min), the specimens were processed in hematoxylin for 3 min, and stained with eosin Y for 45 sec. All sections were incubated in non-xylene solution (*Sigma Chemicals*) for 6 min. After mounted with coverslips, the specimens were examined under a light microscope (*Leica Microsystems*). The assessments were done in a single blinded fashion. 6 to 9 sections were obtained from each tissue block per animal from 6 animals, and 1 section per animal was randomly selected for statistical analysis.

### Lung Injury Assessment

The lung injury scores were estimated based on histological examination, including the hemorrhage of intra-alveolar septa, cell infiltration, and lung wet-to-dry ratio. The scores were determined as 0 (no injury), 1 (10–20% severity), 2 (20–40% severity), 3 (40–50% severity), or 4 (greater than 50% severity) [21]. The degree of intra-alveolar septa hemorrhage was scored as 0 (no hemorrhage), 1 (mild hemorrhage), or 2 (severe hemorrhage). The thickness of intra-alveolar septa was quantified by measuring all septae along a crosshair placed on each image (20 septae for each animal) using Image J software. Lung wet-to-dry ratio that determines pulmonary edema was scored as 0 (no edema), 1 (mild edema), or 2 (severe edema) as compared with the control group.

### Measurement of Survival Rate in Mice

C57BL/6J (B6) mice were used to evaluate survival rate [43]. Our pilot studies demonstrated that LPS at 10 mg/kg (ip) did not cause any death of the mice. At 100 mg/kg (ip), LPS caused approximately 75% of lethality. Furthermore, OroA at 60 mg/kg, but not at 15 mg/kg or 30 mg/kg (ip) improved the survival. This is consistent with the report by others that endotoxin-induced severity and sensitivity is lower in mice than in rats (Parker et al, 2004). For these reasons, higher doses of LPS and OroA were used for studying survival rate in mice. Mice were given lethal dose of LPS (100 mg/kg, ip) to induce endotoxemia, and were divided randomly into 6 groups: (1) *ipSal, 1h or 6h/ipSolv (control) group*: 1 hr or 6 hrs after receiving normal saline (Sal, ip), mice were administered solvent (Solv, ip) (Sal plus Tween 80 at 9∶1 ratio), and examined at different time points for 48 hrs, (2) *ipLPS, 1h or 6h/ipSolv group* **:** 1 hr or 6 hrs after receiving LPS (100 mg/kg, ip), mice were administered Solv (ip) and examined for 48 hrs, (3) *ipLPS, 1h/ipOroA30 group*: 1 hr after LPS (ip) challenge, OroA (30 mg/kg, ip) was administered and examined for 48 hrs, (4) *ipLPS, 1h/ipOroA60 group*: 1 hr after LPS challenge, OroA (60 mg/kg, ip) was administered and examined for 48 hrs, (5) *ipLPS, 6h/ipOroA60 group*: 6 hrs after LPS (ip) challenge, OroA (60 mg/kg, ip) was administered and examined for 48 hrs, and (6) *ipSal, 1h/ipOroA group*: 1 hr after normal saline (Sal, ip) challenge, OroA (30 or 60 mg/kg, ip) was administered and examined for 48 hrs.

### Drugs

OroA was isolated and purified according to our previous report [44] and dissolved in normal saline plus Tween 80 (9∶1 ratio) using ultrasonication. For each experiment, 60 mg/ml stock solution of OroA was diluted to 15 or 30 mg/ml working concentration with normal saline. *E. Coli* LPS was diluted in normal saline to a concentration of 10 mg/ml before use.

### Statistical Analysis

The experimental data were presented as means±SEM. The one-way and two-way ANOVA were used to determine the difference. Measurements at single time point were compared by using the Student’s unpaired t-test. Survival rate were analyzed by Kaplan-Meyer survival curves and Chi-square test was used to test the significance. A *P* value of less than 0.05 was considered statistically significant.
